# Probiotic supplementation in trained trotter horses: effect on blood clinical pathology data and urine metabolomic assessed in field

**DOI:** 10.1152/japplphysiol.01131.2017

**Published:** 2018-04-19

**Authors:** Luca Laghi, Chenglin Zhu, Giuseppe Campagna, Giacomo Rossi, Marilena Bazzano, Fulvio Laus

**Affiliations:** ^1^Centre of Foodomics, Department of Agro-Food Science and Technology, University of Bologna, Cesena, Italy; ^2^Department of Experimental Medicine “Sapienza” University of Rome, Rome, Italy; ^3^School of Biosciences and Veterinary Medicine, University of Camerino, Matelica, Italy

**Keywords:** exercise, lactate, probiotics, Standardbred horse, urine metabolomics

## Abstract

The attention of sports community toward probiotic supplementation as a way to promote exercise and training performance, together with good health, has increased in recent years. This has applied also to horses, with promising results. Here, for the first time, we tested a probiotic mix of several strains of live bacteria typically employed for humans to improve the training performance of Standardbred horses in athletic activity. To evaluate its effects on the horse performance, we measured lactate concentration in blood, a translational outcome largely employed for the purpose, combined with the study of hematological and biochemical parameters, together with urine from a metabolomics perspective. The results showed that the probiotic supplementation significantly reduced postexercise blood lactate concentration. The hematological and biochemical parameters, together with urine molecular profile, suggested that a likely mechanism underlying this positive effect was connected to a switch of energy source in muscle from carbohydrates to short-chain fatty acids. Three sulfur-containing molecules differently concentrated in urines in connection to probiotics administration suggested that such switch was linked to sulfur metabolism.

**NEW & NOTEWORTHY** Probiotic supplementation could reduce postexercise blood lactate concentration in Standardbred horses in athletic activity. Blood parameters, together with urine molecular profile, suggest the mechanism underlying this positive effect is connected to a switch of energy source in muscle from carbohydrates to short-chain fatty acids. Sulfur-containing molecules found in urines in connection to probiotics administration suggested that such switch was linked to sulfur metabolism.

## INTRODUCTION

The attention of sports community toward probiotic supplementation as a way to promote exercise and training performance, as well as good health, has increased in recent years (46, 59). As many as over 700 randomized, controlled, human studies have been conducted with probiotics already in 2011 (56), mainly focusing on gastrointestinal conditions but also included allergic, metabolic, inflammatory, and respiratory conditions. Indeed, in human athletes, probiotic supplementation was found to reduce gut permeability, decrease incidence and severity of respiratory diseases, modulate cytokines production, and increase plasma antioxidant (25, 33, 34, 40, 58, 59). Unfortunately, these studies have been criticized for suggesting a too ample spectrum of incoherent biomarkers of immunological, physiological, and health benefits. Moreover, the studies have been generally found to lack a practical perspective, consisting of translational outcomes or clinical benefits that could be applied by athletes and coaches (46).

Research about probiotics as a way to improve health status, and in turn performance, has focused also on horses, with promising results (1, 21, 24, 48). Unfortunately, the ambiguity of the results seems to affect also the choice of the formulations to be employed in horses, mainly due to the loosely regulated quality of commercial over-the-counter products, together with incoherence in the selection of strains and dosages (50). In addition, many of the formulations increasingly used for horses have been originally designed for humans. This means that any evidence obtained on humans may not be straightforwardly valid for horses. This is specifically true for the genera most commonly used in human probiotic formulations, namely Lactobacillus, Bifidobacterium, and Enterococci, the presence of which in the horse microbiota is limited (11, 13, 14, 50).

In this context, additional research about specific probiotics for use in horses is highly needed for at least three main reasons. First, the safety and absence of side effects of the administration to horses of probiotic formulations originally designed for humans must be assessed. This first safety assessment would allow to by-pass the need for a specific horse formulation reducing the costs of elaboration. Second, it must still be proven whether “human” probiotic strains are able to colonize horses’ intestinal tract. This would confirm the possibility to utilize bacterial strains selected according to their probiotic properties and not their origin (49); third, it must be demonstrated if and to what extent probiotics may influence horses’ training performance. This information is much more limited for horses than for humans (50).

In the present work, we investigated the possibility to use a high concentration multispecies probiotic formulation typically employed for humans to improve the training performance of Standardbred horses. For the purpose, we measured lactate concentration in blood, a translational outcome largely employed for performance. We flanked this observation with the study of urine from a metabolomics perspective, allowing an overall picture of the reaction of the body to exercise (12) and to conditions having direct consequences on the inflammatory status of the body (3, 19), which is often altered by intense exercise (43).

## MATERIALS AND METHODS

### 

#### Ethical issue.

All the described animal-related procedures were conducted according to Directive 2010/63/EU of the European Parliament and of the Council of 22nd September 2010 on the protection of animals used for scientific purposes (Article 1, Paragraph 1, Letter b) and the Italian legislation (D. Lgs. n. 26/2014, Article 2, Paragraph 1, Letter b). The study received the approval of the Ethics Committee of Camerino University (Protocol No. E81AC.8/bis).

#### Animals.

Ten Standardbred horses (4 male and 6 female) were included in the study. The age of the horses ranged between 3 and 8 yr (median 6.5 yr), and the weight was between 405 and 511 kg (median 479 kg). All horses were in activity at the time of the trials and, once enrolled, were stabled in boxes and subjected to a standardized diet composed by hay (a mix of ryegrass and wild oats), complementary feed (containing a mix of oats, barley, wheat, and alfalfa pellets) twice a day (2.5 and 1% of live weight, respectively, divided in 2 rations), and water ad libitum (36). All animals had been dewormed at least 3 mo before the study with a commercial product containing a mix of ivermectine (200 ng/kg body wt) and praziquantel (1.5 mg/kg body wt) and, according to Italian rules, vaccinated against influenza and tetanus. All horses were clinically checked for muscular and respiratory diseases and endoscopically controlled for upper airway dysfunctions.

Animals were clinically checked every day to assess the presence of one of the following conditions causing the exclusion from the study: adverse reaction to the probiotic (e.g., allergy, gastrointestinal symptoms), need for other drugs, dyspnea, anorexia/disorexia, weight loss, nasal discharge, cough, fever, diarrhea, and lameness.

#### Experimental design.

The study was designed as a randomized, double-blinded, placebo-controlled trial with crossover and was conducted in the same racetrack where the horses were housed. The length of the ovoid track was 800 m and made in dry quarry material with the superficial finishing coat in calcareous sand.

Each horse was conducted always by the same driver, using similar standard sulky for competitions.

Before the study, the animals were conditioned for 21 days to daily training sessions of standardized exercise, identical to the training used for the trials described in the following sections. The study was then conducted for 84 days divided in four equal and consecutive training periods lasting 21 days. Each 21-day training period was identical for all horses and consisted of alternated *type A* and *type B* training exercises, following the schedule reported in Table 1 with *exercise B* intended as submaximal effort. Training sessions were always performed in the morning, immediately after consumption of the first food ration of the day.

**Table 1.** Daily exercise of horses for each training session

Type of Exercise	Activities	Day
*Exercise A*	400-m walk 400-m light trot 3,800-m medium trot	1, 2, 4, 5, 6, 8, 9, 11, 12, 13, 15, 16, 18, 19, 20
*Exercise B*	400-m walk 400-m light trot 3,800-m fast trot	3, 7, 10, 14, 17, 21

After 21 days of exercise conditioning, the horses were randomly divided into two groups of five subjects each: *group 1* was treated with food supplemented with Slab51 probiotic mixture for 21 days; and *group 2* was fed with placebo for 21 days. In detail, 17.5 g of probiotic/placebo were administered twice a day directly in the mouth of the horse using a tube dozer.

**Fig. 1.**
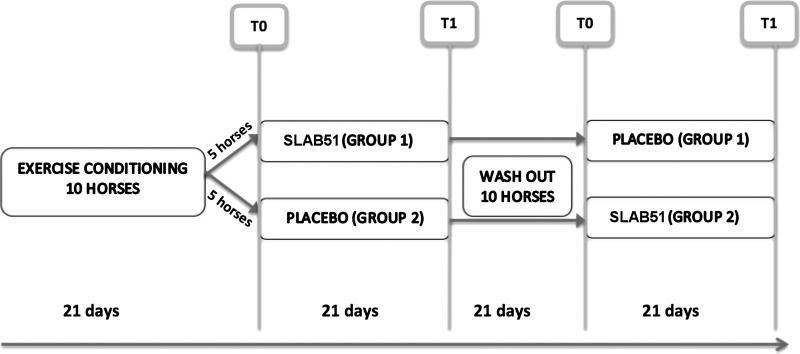
Schedule of the clinical trials activity. T0, before treatment. T1, after treatment.

After a washout period of 21 days, the two groups were reversed so that placebo was administered to *group 1*, and probiotics were administered to *group 2* for an additional 21 days. The activity performed during the clinical trial is illustrated in Fig. 1.

All sampling and clinical evaluations were performed at rest in the morning, 1 h after the end of the feeding, with the exception of lactate evaluation that was performed before starting the exercise and as soon as the horse returned to the box, immediately after the exercise.

#### Probiotic and placebo composition.

The probiotic mixture chosen for the present investigation is a mix of 8 bacterial strains of live lactic acid bacteria and bifidobacteria (probiotic mix Slab51: *Lactobacillus acidophilus* DSM32241, *L. plantarum* DSM32244, *L. casei* DSM 32243, *L. helveticus* DSM 32242, *L. brevis* DSM27961, *Streptococcus thermophilus* DSM 32245, *Bifidobacteria lactis* DSM 32246, and *B. lactis* DSM32247), with the addition of excipients such as maltose and silicon dioxide (Sivoy; Mendes). The placebo was a powder containing only maltose and silicon dioxide as inactive ingredients and was stored in bottles identical to the active product.

#### Sampling of venous blood.

Venous blood samples were collected at rest from the jugular vein into sterile tubes with and without EDTA at T0 (before start of administration) and T1 (at the end of the 21 days of Slab51/placebo administration). With the exception of lactate evaluation, the T1 sampling was performed 12 h after the end of the daily exercise to avoid the effect of the effort on hematological and biochemical parameters (e.g., exercise-induced dehydration, spleen contraction).

The following hematologic parameters were assessed within 12 h from EDTA sample collection, using an hematological cell counter (Cell Dyn 3500, Abbott): erythrocytes (red blood cells), packed cell volume, hemoglobin, red cell distribution width, mean corpuscular volume, mean corpuscular hemoglobin, mean corpuscular hemoglobin concentration, total leukocytes, neutrophils, lymphocytes, monocytes, eosinophils, basophils and platelets, and mean platelet volume. Biochemical profile was assessed on serum samples using a spectrophotometer BT 3000 plus (Biotecnica Instruments, Rome, Italy) and included aspartate aminotransferase, γ-glutamyl transferase, creatinphosphokinase, total protein, albumin, globuline, urea, creatinine, glucose, alkaline phosphstase, lactate dehydrogenase, total bilirubin, direct bilirubin, triglycerides, cholesterol, calcium, phosphorus, magnesium, sodium, potassium, and chlorine.

Blood lactate concentration was tested on whole blood using a portable device (Accutrend Plus; Roche, Mannheim, Germany) both at T0 and T1 immediately before (pre) and after (post) the exercise of the respective day.

#### Urine collection and analysis.

Urine was collected before (pre) and after (post) each training session both at T0 and at T1 during spontaneous urination, using a stick connected with a sterile 50 ml Falcon tube containing sodium azide, directed under the urine flow at the time of urination. Urine was frozen at −80°C within 2 h after collection.

Urine samples were prepared for proton nuclear magnetic resonance (^1^H-NMR) analysis by modifying the protocol described by Barbara et al. (3). Briefly, each sample was centrifuged for 15 min at 15K rpm and 4°C. Then, 0.3 ml of supernatant were added to 0.3 ml of bidistilled water and 0.2 ml of a D_2_O solution of 3-(trimethylsilyl)-propionic-2,2,3,3-d4 acid sodium salt (10 mM). The solution obtained was centrifuged as previously described. ^1^H-NMR spectra were recorded at 298 K with an AVANCE III spectrometer (Bruker, Milan, Italy) operating at a frequency of 600.13 MHz. Following Ventrella et al. (55), the signals from broad resonances originating from large molecules were suppressed by a CPMG filter composed by 400 echoes with a τ of 400 μs and a 180° pulse of 24 μs for a total filter of 330 ms. The HOD residual signal was suppressed by means of presaturation. This was done by employing the cpmgpr1d sequence, part of the standard pulse sequence library. Each spectrum was acquired by summing up 256 transients using 32-K data points over a 7,184-Hz spectral window, with an acquisition time of 2.28 s. By following Bryszewska et al. (5), to apply NMR as a quantitative technique, the recycle delay was set to 5 s, keeping into consideration the relaxation time of the protons under investigation. The signals were assigned by comparing their chemical shift and multiplicity with literature (15), the Human Metabolome Database (60), and Chenomx software library (ver 8.1; Chenomx).

#### Statistical analysis.

Statistical analysis was conducted in R computational language (47). Both pretraining and posttraining, each parameter was influenced on one side by the administration of placebo or treatment (a within subjects variable) and on the other side by the horse (a between subjects variable) (8). The paired nature of the latter variable was taken into consideration by means of a Kruskal-Wallis test for repeated measurements. For the purpose, the aov function of the R package “stats” (7) was applied on the parameters expressed as ranks (10). To circumvent in part the dichotomous application of *P* values, as suggested by Greenland et al. (27) and Wehrens and Franceschi (57), a *P* value trim limit of 0.1 was considered, in agreement with Kang et al. (32).

To partially account for the natural variability of each parameter, the samples obtained at the two T0 points, together with the sample obtained after placebo administration, were considered as describing the animal under no treatment. This choice was based on the rationale that a specific effect ascribable to placebo has never been observed in horses (41). Moreover the mechanism of administration of the probiotic mixture and the fully crossed experimental design were unlikely to lead to any noticeable placebo induced reaction in the animal.

Any correlation among the parameters studied in the present investigation was looked for by calculating their Spearman's rank correlation coefficient, which were considered significant for *P* < 0.05. To remove outliers, a three steps approach was followed. First, each couple of variables was employed to calculate a robust principal component analysis (31) model. Second, the samples’ scores along PC 2 of the corresponding scoreplot were scaled to unity variance. Finally samples were considered as outliers when their score differed more than two standard deviations from the mean.

## RESULTS

No adverse reaction was observed during the trial, and no animal was excluded from the study after clinical and endoscopic assessments.

**Fig. 2.**
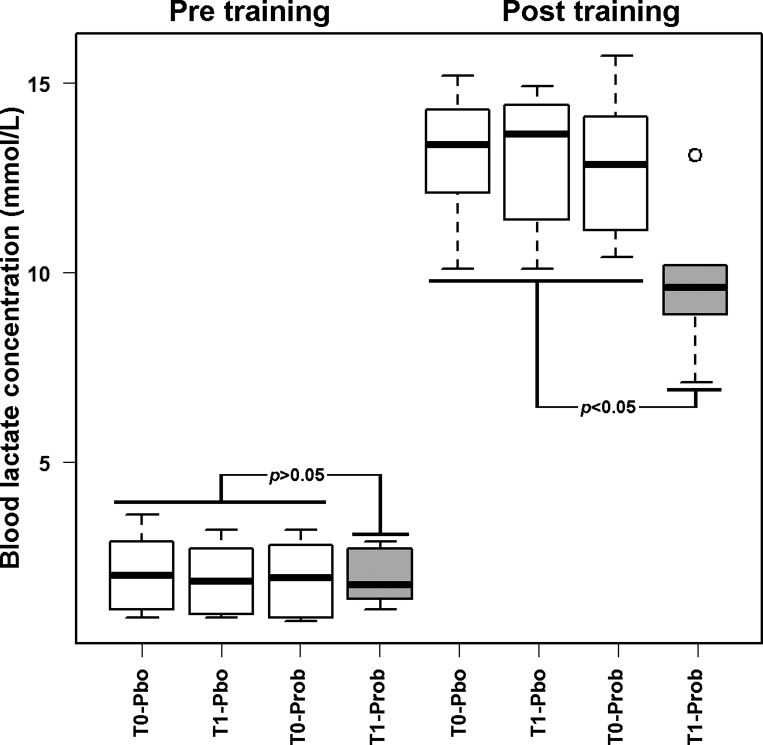
Blood lactate concentration in horses before (T0) or after (T1) treatment with probiotics (Prob) and placebo (Pbo), and pre- and posttraining. Samples obtained at T0 and after treatment with placebo were considered as constituting a single group (white boxplots). Samples obtained after treatment with probiotics (gray boxplots) were compared with them by nonparametric univariate analysis test (*P* values).

To understand whether there was a connection between probiotics administration and resistance of the horses to the effort caused by training, we decided to focus on the concentration of lactate in the blood before and after the daily exercise. Taking into account the natural fluctuations in the concentration of this molecule as a result of postprandial conditions and other confounding factors, we considered the samples obtained at the two T0 points, together with the sample obtained after placebo administration, as describing the animal under no treatment, as outlined in Fig. 2.

Before all training sessions, the horses showed a similar blood lactate concentration, equal to 2.0 ± 0.9 mmol/l. Posttraining, every horse but those at T1 treated with probiotics showed again a similar value of lactate, equal to 13.0 ± 1.8 mmol/l. On the opposite, horses at T1 treated with probiotics showed posttraining a blood lactate concentration of 9.9 ± 1.9 mmol/l, significantly lower than the other groups observed posttraining.

Results concerning hematological and biochemical parameters are reported in Tables 2 and 3, respectively.

**Table 2.** Hematological parameters pretraining

	T0 or T1 Placebo Treatment	T1 Probiotics Treatment	*P*
HCT, %	39.70 ± 4.35	41.20 ± 3.10	0.0692
MCH, pg	16.30 ± 1.33	15.40 ± 1.31	0.0283
MCHC, g/dl	32.80 ± 1.55	32.90 ± 1.71	0.0995
RDWc, %	21.60 ± 0.79	21.20 ± 0.62	0.0452

Values are means ± SD. For readability reasons, only comparisons characterized by a *P* < 0.1 are reported. HCT, hematocrit; MCH, mean corpuscular hemoglobin; MCHC, mean corpuscular hemoglobin concentration; RDWc, red cells distribution width coefficient.

**Table 3.** Biochemical parameters pretraining

	T0 or T1 Placebo Treatment	T1 Probiotics Treatment	*P*
AST, U/l	340.07 ± 57.30	310.40 ± 42.57	0.0913
Albumin, g/dl	3.19 ± 0.27	3.53 ± 0.19	0.0650
Triglycerides, mg/dl	22.07 ± 6.03	21.90 ± 6.52	0.0874
Total calcium, mg/dl	11.14 ± 1.41	11.82 ± 1.45	0.0194
Phosphorus, mg/dl	4.11 ± 1.35	4.93 ± 1.29	0.0547

Values are means ± SD. For readability reasons, only comparisons characterized by *P* < 0.1 are reported. AST, aspartate aminotransferase.

Among hematological parameters, mean corpuscular hemoglobin and red cell distribution width coefficient showed lower values for samples obtained at the end of probiotics administration with high significance (*P* < 0.05). Two other parameters, hematocrit and mean corpuscular hemoglobin concentration, had higher values at the end of probiotics administration in the horses considered but only with scarce significance (*P* < 0.1). Among biochemical parameters, only calcium concentration showed highly significant differences (*P* < 0.05) between the two identified groups of samples.

The investigation of the impact of probiotics supplementation was then extended to urine metabolome before the daily exercise. The molecules that could be quantified pertained mainly to the chemical groups of amino acids, short chain fatty acids, organic acids, and monomeric carbohydrates (15). The molecular profile for probiotics at T1 differed from the others in the concentration of eight molecules (Table 4).

**Table 4.** Concentration of the molecules with concentration pretraining varying because of probiotic supplementation

	T0 or T1 Placebo Treatment	T1 Probiotics Treatment	*P*
2-Hydroxyisovalerate	1.83 × 10^−2^ ± 1.64 × 10^−2^	1.37 × 10^−2^ ± 1.35 × 10^−2^	0.0469
*Trans*-aconitate	3.09 × 10^−1^ ± 2.45 × 10^−1^	1.67 × 10^−1^ ± 7.38 × 10^−2^	0.0451
Citrate	1.25 × 10^−1^ ± 2.43 × 10^−1^	9.71 × 10^−2^ ± 4.13 × 10^−2^	0.0733
*P*-cresol sulfate	3.60 × 10^−2^ ± 1.72 × 10^−2^	2.79 × 10^−2^ ± 1.81 × 10^−2^	0.0756
Dimethyl sulfone	3.90 × 10^−1^ ± 2.64 × 10^−1^	5.05 × 10^−1^ ± 3.03 × 10^−1^	0.0462
Glycine	8.28 × 10^−1^ ± 1.47	2.93 × 10^−1^ ± 1.16 × 10^−1^	0.0204
Pantothenate	1.60 × 10^−1^ ± 3.61 × 10^−2^	1.72 × 10^−1^ ± 3.83 × 10^−2^	0.0550
Taurine	3.23 ± 2.23	2.16 ± 1.38	0.0036

Values are means ± SD (in mM). For readability reasons, only comparisons characterized by *P* < 0.1 are reported.

**Fig. 3.**
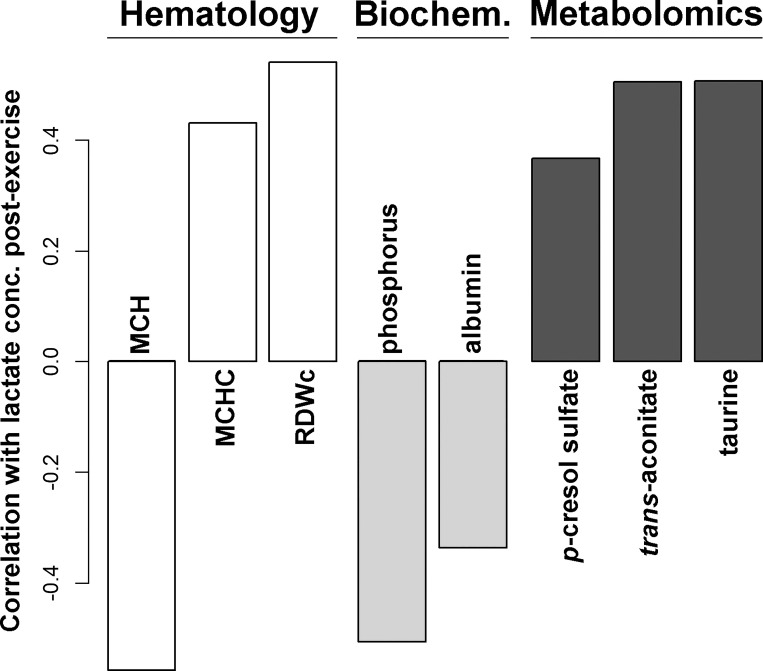
Significant correlations (*P* < 0.05) with lactate concentration registered postexercise of the hematological or biochemical parameters and the molecules listed in Tables 2, 3, and 4. MCH, mean corpuscular hemoglobin; MCHC, mean corpuscular hemoglobin concentration; RDWc, red cells distribution width coefficient.

Figure 3 reports the hematological or biochemical parameters and the molecules listed in Tables 2, 3, and 4 that showed a correlation with lactate concentration registered postexercise.

## DISCUSSION

The specific probiotic formulation used in this study did not cause any adverse reactions in the horses. Animals were daily monitored throughout the study, and no clinically evident side effect was registered. Furthermore, the changes found in hematological and biochemical parameters cannot be associated with modifications of the metabolism, which would negatively affect the sanitary status of the animal. It can therefore be concluded that the tested probiotic formulation does not cause any detrimental effect and can be safely used in horses.

Blood lactate response to exercise can be used in field test for assessing performance and fitness, because it reflects the reduction of lactic acid production and accumulation in muscular cells, with direct consequences on the onset of fatigue in athletic horses (12, 24, 48). Postexercise, the blood lactate concentration was statistically lower in horses treated with probiotics than in the other groups. Lactate accumulation in muscles can lead to impairment of glycolysis and respiratory capacity of mitochondria (the main source of plasmatic AST) (30), decreasing in ATP concentration and availability, and to sarcoplasmic reticulum swelling (29, 53). These mechanisms are the basis of the onset of fatigue in athletic horses (28). This is why lactate concentration in blood, reflecting the one in muscles, is largely employed as an index of performance, also because it is considered as more reliable than oxygen consumption rate and heart beat (26). Lactate accumulation is usually evaluated by measuring the VLa4 (the speed at lactate concentration of 4 mmol/l): as the horse increases fitness, VLa4 increases. However, the blood lactate response to a single episode of submaximal exercise can be used as an alternative (16) to such method. Since a lower blood lactate concentration has been found in the treated group after exercise, probiotics seem to promote the performance of the athletic horses, which could be correlated with better performance during the race.

Several mechanisms have been described that could account for this finding, mainly focusing on the possibility for the muscles to use short-chain fatty acids (SCFAs) instead of carbohydrates as energy source. Lactobacilli supplementation can modify hindgut pH and induce the proliferation of other genus such as *Veillonella* spp., the most abundant lactate utilizing bacteria in horse gut (4), modifying the energy source during exercise. In a previous report, unconditioned horses supplemented with yeast exhibited lower plasma lactate concentration than unsupplemented horses (24). The authors speculated that probiotics administration could modify gut fermentation and, consequently, could increase the amount of circulating SCFAs, which are efficiently used as energy source in exercise. These findings are supported by the results obtained by Garcia et al. (21), who found that horses receiving probiotics better digested hemicelluloses. In a previous work, Medina et al. (42) showed that probiotics such as *Saccharomyces cerevisiae* are able to modify production and proportion of SCFAs in the large intestine. This could make the horses use a lower amount of carbohydrates, which would account for the lower production of muscular lactic acid observed in the present study. In horses receiving probiotic, Art et al. (1) found that carbohydrate aerobic enzymatic capacity and carbohydrate utilization are improved. Furthermore, oral administration of *Lactobacillus plantarum* showed to have a potential for the removal and utilization of blood lactate after exercise in mice (9).

The idea of a profile modification of the molecules employed by the horse muscles as energy source seems to be supported, in the present paper, by the metabolomics observation of urine preexercise. The concentration of *trans*-aconitate, in particular, was found to be significantly modified by the administration of probiotics and was found to correlate with lactate concentration in blood postexercise. *Trans*-aconitate offers an insight into TCA cycle, as this molecule is endogenously originating from the cycle’s intermediates, through *cis*-aconitate, by means of *trans*-aconitate decarboxylase (23). As a confirmation, the concentration of *trans*-aconitate in human urines proved to be modified by exercise sessions (45).

Citrate concentration trend pairs the one of *trans*-aconitate in giving information about TCA cycle efficiency in the horses under investigation. Indeed, the citrate concentration in urine can be considered as an indirect biomarker of the horses training status. In fact, endurance training is known to modulate the concentration in mitochondria of citrate synthase enzyme (51), which in turn modulates the concentration of TCA intermediates. Morevoer, evidence gathered in humans shows that citrate is excreted with urines at lower levels in trained subjects (37).

The trend evidenced for triglycerides concentration in blood seems to offer a different prospective of the same phenomenon. In fact, the decreasing trend of triglycerides in blood connected to probiotics supplementation could also be attributed to the accumulation of SCFAs that pass from the lumen into the bloodstream regulate the balance in fatty acids synthesis (6, 9, 22). Indeed, a similar effect of *Lactobacillus plantarum* has also been found in experimental exercise mice and other laboratory animals (9, 44) and could also occur in horses.

To better characterize the underlying reasons for the energy switch that seems to be observed in the present investigation, urine taurine, *p*-cresol sulfate, and dimethyl sulfone offer a compelling clue for reflection. Among the molecules characterized by urine metabolomics, these are the only ones that contain sulfur and their concentration was found to be altered by probiotic administration. Moreover, *p*-cresol sulfate and taurine have also been found positively correlated to lactate concentration in blood postexercise, as shown in Fig. 3. These molecules seem therefore to suggest that sulfur metabolism may play a role in the highlighted energy metabolism changes. These molecules are actually noteworthy per se in the present context. The nonproteogenic amino acid taurine plays a pivotal role in skeletal muscle development (38) and high excretion of it through urine has even been associated to disuse-related muscle atrophy. *P*-cresol sulfate seems to be doubly linked to body inflammatory status, so that its low concentration in urine seems to be desirable. *P*-cresol sulfate is found in urine solely as a result of colon bacteria catabolism of food components that escape digestion (17). In particular, its production has been linked to disordered bacterial colonization in inflammatory bowel disease (20). Despite the inconclusive and sometimes misunderstood literature (54), *p*-cresol sulfate is considered as exerting a toxic effect on the body at several levels, thus leading to systemic inflammation (35). Dimethyl sulfone has been found to protect horses from systemic inflammation connected to exercise injuries (39), probably by exerting a scavenging effect on reacting oxygen species, so that it may even be employed as a horse food additive.

Other parameters were statistically influenced by probiotics administration, although not clinically relevant, such as calcium and phosphorus with trends linked to SCFAs production, which can modulate both their release and absorption (52). The lower detection of aspartate aminotransferase in the probiotic group after exercise suggests a beneficial effect of probiotics on muscular cell preservation, due to the controlled intracellular lactic acidosis under the effects of the mechanisms previous described (6, 9, 18).

The link between gut microbiota and catecholamines has been demonstrated in mice (2) and, in the author’s opinion, the significant hematocrit increase in the probiotic group should be considered related to a more vigorous adrenergic effect caused by catecholamines production, as suggested by other authors (61). The rise in hematocrit leads to an increase of red cells number in bloodstream and could therefore contribute to a better oxygenation of tissues and, consequently, to a more proficient utilization of aerobic pathways.

### 

#### Conclusion.

The results of the present study show that probiotic supplementation could reduce postexercise blood lactate concentration in Standardbred horses in athletic activity.

The tested hematological and biochemical parameters, along with urine molecular profile, suggest that a likely mechanism underlying this positive effect is connected to a switch of energy source in muscle from carbohydrates to SCFAs.

A possible limitation of the study could be the lack of direct information about the real colonization of the horse gut by the probiotic used. Further studies could better clarify this issue.

## GRANTS

C. Zhu gratefully acknowledges financial support from Chinese Scholarship Council (Grant 201606910076).

## DISCLOSURES

No conflicts of interest, financial or otherwise, are declared by the authors.

## AUTHOR CONTRIBUTIONS

F.L. conceived and designed research; G.C. and F.L. performed experiments; L.L., C.Z., and F.L. analyzed data; L.L., C.Z., G.R., M.B., and F.L. interpreted results of experiments; L.L., C.Z., and F.L. prepared figures; L.L., C.Z., G.R., and F.L. drafted manuscript; L.L., C.Z., G.R., M.B., and F.L. edited and revised manuscript; L.L., C.Z., G.C., G.R., M.B., and F.L. approved final version of manuscript.
